# A Review of Treatment and Prevention of Coronavirus Disease 2019 among Solid Organ Transplant Recipients

**DOI:** 10.3390/v13091706

**Published:** 2021-08-27

**Authors:** Deanna J. Buehrle, Robert R. Sutton, Erin L. McCann, Aaron E. Lucas

**Affiliations:** 1 Department of Medicine, Veterans Affairs Pittsburgh Healthcare System, Pittsburgh, PA 15240, USA; aaron.lucas@va.gov; 2 Department of Pharmacy, Veterans Affairs Pittsburgh Healthcare System, Pittsburgh, PA 15240, USA; robert.sutton@va.gov (R.R.S.); erin.mccann@va.gov (E.L.M.)

**Keywords:** solid organ transplant, COVID-19, vaccine, SARS-CoV-2, immunosuppressed

## Abstract

Therapeutic management of solid organ transplant (SOT) recipients infected with severe acute respiratory syndrome coronavirus 2 (SARS-CoV-2), the cause of coronavirus disease 2019 (COVID-19), may challenge healthcare providers given a paucity of clinical data specific to this cohort. Herein, we summarize and review the studies that have formed the framework for current COVID-19 consensus management guidelines. Our review focuses on COVID-19 treatment options including monoclonal antibody products, antiviral agents such as remdesivir, and immunomodulatory agents such as corticosteroids, interleukin inhibitors, and kinase inhibitors. We highlight the presence or absence of clinical data of these therapeutics related to the SOT recipient with COVID-19. We also describe data surrounding COVID-19 vaccination of the SOT recipient. Understanding the extent and limitations of observational and clinical trial data for the prevention and treatment of COVID-19 specific to the SOT population is crucial for optimal management. Although minimal data exist on clinical outcomes among SOT recipients treated with varying COVID-19 therapeutics, reviewing these agents and the studies that have led to their inclusion or exclusion in clinical management of COVID-19 highlights the need for further studies of these therapeutics in SOT patients with COVID-19.

## 1. Introduction

Management and prevention of Coronavirus disease 2019 (COVID-19), which is caused by severe acute respiratory syndrome coronavirus 2 (SARS-CoV-2), can be challenging among solid organ transplant (SOT) recipients. Frequent healthcare visits, the presence of medical comorbidities, and modulated host immune response due to immunosuppressive medication place SOT recipients at high risk of contracting SARS-CoV-2 infection and developing severe COVID-19.

Mortality rates reported among SOT recipients with COVID-19 vary widely from 3% to over 30% depending on organ type, comorbid conditions, geographical location, COVID-19 treatment, and time of report during the pandemic [1,2]. Data are conflicting as to whether SOT recipients have higher risk of mortality compared with non-SOT recipients [2,3,4,5,6,7,8]. Furthermore, SOT recipients often have comorbidities, such as diabetes, chronic kidney disease, and cardiac disease, that have been associated with more severe cases of COVID-19 and an increased risk of mortality. As such, it is difficult to determine the attributable impact of SOT recipient status on COVID-19 morbidity and mortality.

During the COVID-19 pandemic, scientific efforts have advanced therapeutic options and vaccine development. In some cases, these therapeutics have halted the progression of COVID-19 or have improved survival. In this review, we summarize the data surrounding treatment options for COVID-19, with a focus on data specific to SOT recipients, including literature on vaccination against COVID-19 in SOT recipients. Throughout this review, severity of illness categories are defined in accordance with National Institutes of Health (NIH) COVID-19 Treatment Guidelines [9]. Mild illness is defined as signs and symptoms of COVID-19 without an oxygen saturation (SpO2) <94% on room air, dyspnea or abnormal chest imaging, moderate illness is defined as evidence of lower respiratory disease clinically or on imaging with an SpO2 ≥94% on room air, severe illness is defined as an SpO2 <94% on room air, and critical illness is defined as respiratory failure, septic shock, or multiple organ dysfunction.

## 2. Basic Principles 

### 2.1. Virology

Fundamental concepts of both SARS-CoV-2 virology and host immune response during infection illuminate therapeutic targets for antiviral and immunomodulating drugs. Viral transmission of SARS-CoV-2, an enveloped, positive-sense, single-stranded ribonucleic acid (RNA) betacoronavirus, primarily occurs through inhalation of infected respiratory droplets, leading to an interface of SARS-CoV-2 with human respiratory tract and alveolar epithelial cells, vascular endothelial cells, and alveolar macrophages [10]. SARS-CoV-2 spike glycoprotein (S protein) then binds to the ACE2 receptor of target host cells in synergy with the host transmembrane serine protease 2, allowing membrane fusion and release of the viral genome into host cytoplasm through an endocytic pathway [11]. Following host cell entry, the virus is disassembled to release nucleocapsid and viral genome, which host ribosomes translate into polyproteins [11]. Proteases cleave polyproteins into structural and non-structural proteins that include the replication-transcription complex, containing the RNA-dependent RNA polymerase that is highly conserved and critical to the SARS-CoV-2 life cycle by mediating transcription and replication of the RNA genome [11]. The process concludes with assembly of virion components into the endoplasmic reticulum Golgi intermediate compartment complex, virus maturation, and release from infected cells by exocytosis [11]. This leads to infection of neighboring cells and viremia. Importantly, inhibitors of messenger RNA (mRNA) translation have demonstrated in vitro antiviral activity [12]. 

### 2.2. Immunology

Morbidity and mortality associated with COVID-19 often relate to an inflammatory phase following infection, respiratory failure from acute respiratory distress syndrome (ARDS), and a cytokine storm-like syndrome. Once SARS-CoV-2 enters its host cells, active replication and release of the virus causes a highly inflammatory programmed cell death [13]. Unlike many other respiratory viral infections, interferon (IFN)-1 and IFN-III response is reduced in COVID-19, dampening early host innate antiviral activity [14]. Pathogen-associated molecular patterns and damage-associated molecular patterns are released and recognized by neighboring alveolar epithelial cell and alveolar macrophage pattern-recognition receptors [13]. This process activates down-stream proinflammatory transcription factors, leading to production of chemokines and cytokines that recruit monocytes, NK cells, dendritic cells, and lymphocytes [13,14].

A protective immune response involves early rapid clearance of infected cells, functional CD4 T cell and B cell activity, and a humoral response with development of neutralizing IgA, IgM, and IgG antibodies [13,14]. Conversely, a dysfunctional immune response may develop with a suppressed Th1 adaptive antiviral immune response, virus-specific CD8 T cell and NK cell exhaustion and dysfunction, excessive infiltration of monocytes, macrophages, and T cells, systemic CRS, pulmonary edema and pneumonia, and widespread inflammation and multi-organ damage [13,14,15]. It is hypothesized that an early CD4 and CD8 T cell response is protective, but a late T cell response, caused by SARS-CoV-2 immune evasion, may amplify pathogenic inflammation in the setting of high viral load. Of note, high levels of IL-6 have been associated with severe disease and poor prognosis [16]. Th17-associated cytokines have also been associated with further release of proinflammatory cytokines and ARDS [15].

Immunomodulating drugs that can limit the hyperinflammatory pathogenic cellular and humoral immune response caused by SARS-CoV-2 infection are being investigated and implemented into the clinical management of patients with COVID-19. However, SOT-related immunosuppressing drugs further complicate the immunopathogenesis and the role of some immunomodulating drugs under investigation for COVID-19 treatment.

## 3. Therapeutics

### 3.1. Monoclonal Antibodies

Anti-SARS-CoV-2 monoclonal antibodies bind to epitopes of the S protein receptor-binding domain of SARS-CoV-2. This neutralizing action prevents S protein-dependent viral entry by inhibiting receptor-binding domain interaction with ACE2 on host cells [16]. Neutralizing monoclonal antibodies were first derived from convalescent plasma of patients infected with SARS-CoV-2 [17]. In animal models, several products were found to decrease viral load and reduce infection-related lung damage when given prophylactically and therapeutically [16,18]. To date, five monoclonal antibody products, bamlanivimab, etesevimab, casirivimab, imdevimab, and sotrovimab, have been developed for clinical use in humans.

The Blocking Viral Attachment and Cell Entry with SARS-CoV-2 Neutralizing Antibodies (BLAZE) Trials are a series of company sponsored trials of bamlanivimab and etesevimab. The BLAZE-1 study was a double-blind RCT with three phases of outpatients with mild or moderate COVID-19. During the first phase (randomization to one of three doses of bamlanivimab or placebo), patients who received the 2800 mg dose had a significantly higher decrease in viral load than patients receiving placebo by day 11, and patients who received the study drug had less hospitalizations than those receiving placebo [19]. A post hoc analysis of high-risk patients (age ≥65 years, obesity, or prespecified coexisting conditions such as immunosuppressed status) revealed lower hospitalization rates among those receiving bamlanivimab versus placebo (4% versus 15%). Based on this data, an emergency use authorization (EUA) of bamlanivimab was granted by the Food and Drug Administration (FDA) for outpatients meeting certain criteria (including immunosuppressive disease or receipt of immunosuppressive medications) with mild to moderate COVID-19. During the second phase, patients were randomized to receive bamlanivimab alone, bamlanivimab plus etesevimab, or placebo [20]. Compared with placebo, only the combination of bamlanivimab and etesevimab resulted in a significant difference in the change in viral load at day 11, however, all study drug arms had numerically less hospitalizations than the placebo arm. During the BLAZE-1 Phase 3 trial that only included patients with high-risk conditions, there were significantly less hospitalizations and deaths in the group receiving combination therapy versus placebo [21]. Full data from the BLAZE-1 Trial, in addition to data from the ongoing BLAZE-4 Trial, were evaluated by the FDA and an EUA of bamlanivimab and etesevimab was granted in February 2021 for patients with mild to moderate COVID-19 with high risk of progression. The EUA of bamlanivimab alone has been revoked and the combination is no longer recommended due to concerns for reduced susceptibility of variants to these agents as of August 2021.

Casirivimab, etesevimab, and sotrovimab appear to retain activity against most currently circulating variants. The antibody cocktail REGN-COV2, a combination of casirivimab and imdevimab, is currently being evaluated in phase 1 to 3, double-blind, randomized, placebo-controlled trials. Per an interim report, outpatients with COVID-19 were randomized to receive 2.4 g of REGN-COV2, 8.0 g of REGN-COV2, or placebo [22]. In the combined REGN-COV2 dose group, there was a significantly higher decrease in viral load from day 1 through day 7 versus placebo, especially among seronegative patients. Data from these trials were evaluated by the FDA and an EUA of casirivimab and imdevimab for the treatment of outpatients with mild or moderate COVID-19 at high risk of progression was also granted. Similarly, sotrovimab was recently granted an EUA by the FDA based on ongoing data from a phase 3 trial demonstrating lower need for hospitalization in outpatients given the drug versus placebo [23]. 

History of SOT was considered a risk factor for hospitalization in the aforementioned clinical trials. Indeed, a monitoring program of outpatient kidney transplants with COVID-19 in New York City noted that 34 out of 44 were ultimately referred to the emergency department and required hospitalization; 6 died [24]. Although outcomes in monoclonal antibody trials are not stratified by SOT recipient status specifically, they appear to be better among patients with a risk factor for hospitalization who receive monoclonal antibodies versus placebo. Importantly, monoclonal antibody reduced viral load to a greater degree among patients who did not yet have an endogenous immune response [22]. Real-world data surrounding the use of monoclonal antibody among SOT recipients is sparse. In an observational study comparing outpatients who received bamlanivimab with non-treated patients, bamlanivimab was associated with fewer hospitalizations and mortality within 28 days [25]. This study included 12 SOT recipients but outcomes were not reported specific to this group. Taken together, these data highlight the need for early intervention to prevent progression of COVID-19 among SOT recipients. Monoclonal antibodies are appealing options for this population, which may have a delayed or inadequate endogenous immune response. As such, these agents are currently recommended in some consensus guidelines for non-hospitalized patients who are at high risk of disease progression as defined by FDA EUA criteria (Table 1). 

### 3.2. COVID-19 Convalescent Plasma

Convalescent plasma (CP) contains anti-SARS-CoV-2 antibodies collected from human donors with prior COVID-19. In April 2020, the National Expanded Access Program (EAP) for CP was started. The FDA evaluated early data from the EAP, along with data from two underpowered RCTs, and concluded that CP met the “may be effective” criterion and issued an EUA for its use in August 2020 [26,27].

After the issuance of the EUA, data from several early RCTs were made available. These studies, which randomized patients hospitalized for COVID-19 or patients with severe COVID-19 to CP or placebo, showed no difference in progression to severe disease or death between arms [28,29,30]. There were two main limitations to these studies. First, many subjects in the PLACID study received low titer CP. Second, in each of the studies, the median symptom duration prior to enrollment was 8 days and many subjects had detectable antibodies prior to receiving CP. The RECOVERY Trial, the largest study of CP to date, randomized over 5000 hospitalized patients in each arm to receive either CP or usual care [31]. There was no difference in 28-day mortality (24% in each arm), the proportion of patients discharged at 28 days, or progression to mechanical ventilation. The 28-day mortality rate ratio was similar for all subgroups including those without antibodies at baseline. However, as observed in the previous trials, the median time from symptom onset was 9 days.

**Table 1:** Characteristics of Proposed Therapies for the Treatment of COVID-19.2022.

Class and Agent	Dosing	● Place in Therapy	Drug-Drug Interactions	Adverse Effects	Special Considerations for Transplant Recipients
**Antivirals**					
Remdesivir	200 mg IV for 1 d, followed by 100 mg for 5–10 d	**NIH Guidelines [9]:** Recommended for pts hospitalized and requiring supplemental oxygen (alone BIIa, with dexamethasone BIII) Recommended for pts hospitalized and requiring high-flow oxygen or noninvasive ventilation with dexamethasone (BIII) **WHO Guidelines [32]:** Recommend against remdesivir in addition to usual care for any disease severity (conditional recommendation against) **IDSA Guidelines [33]:** Recommended for pts hospitalized with SpO2 ≤94% on room air, requiring supplemental oxygen, mechanical ventilation, or ECMO (5 d course) (conditional recommendation, moderate certainty of evidence)	Substrate of CYP3A4, OATP1B1, and P-gp and an inhibitor of CYP3A4, OATP1B1, OATP1B3, and MATE1 Hydroxychloroquine and chloroquine may diminish the effects of remdesivir; combined use is not recommended Formal drug interactions studies have not been conducted	Bradycardia Hypotension Increased serum ALT and AST Hypersensitivity reactions Prolonged prothrombin time Nausea	Monitor closely for drug interactions Not recommended if eGFR <30 mL/min due to concern for vehicle (SBECD) accumulation leading to renal or liver injury, however, toxicity was not observed in a retrospective study [34] Discontinue if ALT levels increase to >10 times the upper limit of normal, or if ALT elevation with signs/symptoms of liver injury Most patients should receive a 5 d course; no difference in outcomes with 10 d vs. 5 d
Chloroquine or Hydroxychloroquine (with or without azithromycin)	Hydroxychloroquine ^a^: 800 mg q6h for 2 doses, followed by 400 mg q12h for 9 days or until discharge Chloroquine: 500 mg PO q12h for 7–14 d Azithromycin: 500 mg on d 1, then 250 mg once daily on d 2–5 or 500 mg once daily for 7 d	**NIH Guidelines:** Recommend against use in hospitalized pts (AI) Recommend against use in nonhospitalized pts, except in clinical trial (AIIa) **WHO Guidelines:** Strongly recommend against for prophylaxis or treatment of COVID-19 (recommendation against) **IDSA Guidelines:** Recommend against use in hospitalized pts (strong recommendation, moderate certainty) Recommend against use in nonhospitalized pts (strong recommendation, low certainty)	Minor substrate of CYP2D6 QT prolongation with other medications that prolong QT interval May decrease therapeutic effects of remdesivir; avoid combination Increased risk of hemolytic reactions with dapsone May increase levels of digoxin and cyclosporin	QT prolongation Cardiomyopathy Hypersensitivity reactions Hypoglycemia Myopathy Neuropsychiatric effects Retinopathy	Should not be used for hospitalized patients with COVID-19
Ivermectin	100–400 µg/kg daily for up to 5 d	**NIH Guidelines:** Insufficient data to recommend for or against use for the treatment of COVID-19 **WHO Guidelines:** Recommend against for treatment of COVID-19 outside of a clinical trial (only in research setting) **IDSA Guidelines:** Recommend against use in nonhospitalized and hospitalized pts outside of a clinical trial (conditional recommendation, very low certainty)	Minor CYP3A4 and P-glycoprotein substrate	Mazzotti reaction associated with onchocerciasis Cardiovascular (tachycardia, edema) Dizziness Gastrointestinal upset AST/ALT elevations Blood dyscrasias	May be used in patients with COVID-19 receiving immunosuppressive therapy from countries with high prevalence of strongyloidiasis as antihelminthic
Lopinavir/ritonavir	400 mg/100 mg PO q12h for 7–14 d	**NIH Guidelines:** Recommend against use for nonhospitalized (AIII) and hospitalized (AI) pts **WHO Guidelines:** Recommend against for treatment of COVID-19 (recommendation against) **IDSA Guidelines:** Recommend against use in hospitalized pts (strong recommendation, moderate certainty)	Extensive and significant drug interactions exist; review of full drug regimen is recommended prior to use Strong CYP3A4 inhibitor; concentrations of drugs metabolized by CYP3A4 may be increased Anticoagulants Antiplatelet Azole antifungals Calcineurin inhibitors	Gastrointestinal upset Hepatotoxicity Dermatologic Endocrine and metabolic abnormalities CNS adverse effects	Should not be used in the treatment of COVID-19
**Anti-SARS-CoV-2 Antibody Products**					
Monoclonal Antibodies	Bamlanivimab 700 mg plus etesevimab 1400 mg IV as a single dose Casirivimab 600 mg plus imdevimab 600 mg IV/SQ as a single dose Sotrovimab 500 mg IV as a single dose	**NIH Guidelines:** Casirivimab plus imdevimab or sotrovimab recommended for outpatients with mild to moderate COVID-19 who are high-risk as defined by EUA criteria (AIIa for casirivimab plus imdevimab) Recommend against use of bamlanivimab plus etesevimab (AIII) Recommend against use in hospitalized patients outside of a clinical trial (AIIa) **WHO Guidelines:** Class not addressed **IDSA Guidelines:** Recommended for ambulatory pts with mild to moderate COVID-19 at high risk for progression to severe disease (conditional recommendation, low certainty)	May decrease the effects of COVID-19 vaccine; postpone administration of COVID-19 vaccine until at least 90 days after treatment	Hypersensitivity reactions Pruritis Injection site reactions Fever	Authorized under FDA EUA Administer in healthcare settings by qualified healthcare professional with access to medications to treat infusion reactions Monitor patient for at least 1-h post-administration Use of bamlanivimab alone and bamlanivimab plus etesevimab is not recommended due to decreased susceptibility of SARS-CoV-2 variants
Convalescent Plasma (CP)	1 unit (approximately 200 mL) of high-titer ^b^ CP IV as a single dose; an additional unit may be considered based on prescriber judgement	**NIH Guidelines:** For hospitalized pts without impaired immunity: recommend against use (AI) For hospitalized pts with impaired immunity: insufficient data to recommend for or against use of high-titer CP For nonhospitalized pts: insufficient data to recommend either for or against use outside of a clinical trial Recommend against use of low-titer CP in any setting **WHO Guidelines:** Class not addressed **IDSA Guidelines:** Recommend against use for hospitalized pts (conditional recommendation, low certainty) Recommend only in the context of a clinical trial for ambulatory pts with mild to moderate COVID-19 (knowledge gap)	May decrease the effects of COVID-19 vaccine; postpone administration of COVID-19 vaccine until at least 90 days after treatment	Transfusion reactions	Authorized under the EUA for the treatment of hospitalized patients with COVID-19 and impaired immunity Careful history should be taken for previous transfusion reactions Monitor vital signs before, during, and after infusion Patients with cardiac disease may require lower volume and slower infusion Low-titer CP should not be used
**Immunomodulators**					
Corticosteroids	Dexamethasone: 6 mg IV/PO q24h for 10 d or until hospital discharge Equivalent daily doses: Prednisone 40 mg Methylprednisolone 32 mg Hydrocortisone 160 mg	**NIH Guidelines:** Recommended for pts hospitalized and requiring supplemental oxygen (alone BI, with remdesivir BIII) Recommended for pts hospitalized and requiring high-flow oxygen or noninvasive ventilation (alone AI, with remdesivir BIII) Recommended for pts hospitalized and requiring mechanical ventilation or ECMO (AI) **WHO Guidelines:** Recommended for pts with severe or critical COVID-19 (recommended) Recommended against use for pts with non-severe COVID-19 (conditional recommendation against) **IDSA Guidelines:** Recommended for pts hospitalized with SpO2 ≤94% on room air or requiring supplemental oxygen (conditional recommendation, moderate certainty) Recommended for pts hospitalized on mechanical ventilation or ECMO (strong recommendation, moderate certainty) Recommend against use for pts with SpO^2^ >94% not requiring supplemental O_2_ (conditional recommendation, low certainty)	Major substrate of CYP3A4 Minor substrate of P-gp/ABCB1 Weak inducer of CYP3A4 May decrease the concentration of tacrolimus	Immunosuppression Adrenal insufficiency and suppression Psychiatric disturbances Gastrointestinal issues (increased appetite, peptic ulcers, esophagitis)	Risk of reactivation of latent infections such as strongyloidiasis, HBV, HSV, TB Monitor closely for new secondary infections Co-management of immunosuppression for transplant recipients and COVID19 therapy necessitates a careful balance of minimizing maintenance medications and utilizing evidence based primary treatment Corticosteroids have historically been mainstays of maintenance immunosuppression for transplant recipients and when used to manage pulmonary manifestations of SARS-CoV-2, may also serve as prophylaxis against allograft rejection
Immunoglobulins	500 mg/kg daily for 5 d	**NIH Guidelines:** Recommend against use of non-SARS-CoV-2-specific IVIG for COVID-19 outside of a clinical trial (AIII) **WHO Guidelines:** Agent not addressed **IDSA Guidelines:** Agent not addressed	May interfere with response to COVID-19 vaccination	Thrombosis Hemolysis Renal failure Hypertension Transfusion-related lung injury Flu-like symptoms	Monitor closely for transfusion-related reactions Use of IVIG is appropriate if being used for other indications during COVID-19 illness
Interleukin-6 Inhibitors	Tocilizumab: 8 mg/kg (maximum 800 mg) IV as a single dose	**NIH Guidelines:** Either baricitinib or tocilizumab is recommended in conjunction with dexamethasone in pts within 3 d of hospitalization who have rapid respiratory decompensation (admitted to ICU within 24 h and require mechanical ventilation, noninvasive ventilation, or high-flow, or have rapidly escalating O2 needs and require noninvasive ventilation or high-flow with a CRP ≥ 75 mg/L) (BIIa) Insufficient evidence to recommend for or against use in hospitalized pts on conventional O2 **WHO Guidelines:** Agent not addressed **IDSA Guidelines:** Suggested for pts with progressive severe or critical COVID-19 who have elevated inflammatory markers (i.e., CRP ≥75 mg/L) with corticosteroid (conditional recommendation, low certainty)	May enhance immunosuppressive effects with other immunosuppressive medications May interfere with response to COVID-19 vaccination	Neutropenia Hepatotoxicity Infusion-related reactions Hypertension Headache	The safety of IL-6 inhibitors is unknown in patients who are significantly immunosuppressed; the NIH guidelines recommend against use in this population Serious infections (fungal, bacterial, TB, viral, opportunistic) have occurred in patients receiving long courses of IL-6 inhibitors Consider ivermectin in patients who are receiving tocilizumab and corticosteroid in areas where strongyloidiasis is endemic Drug labeling recommends to avoid in ANC <2000/mm^3^, plt <100,000/mm^3^, and AST or ALT >1.5 times ULN
Interleukin-1 Inhibitors	Anakinra 100 mg SQ q12h for 72 h, followed by 100 mg SQ daily for 7 d	**NIH Guidelines:** Insufficient evidence to recommend for or against use **WHO Guidelines:** Agent not addressed **IDSA Guidelines:** Agent not addressed	Additive immunosuppression when used with other immunosuppressive agents	Flu-like symptoms Injection site reactions Gastrointestinal upset Hepatoxicity Anaphylaxis	Avoid in patients on TNF-alpha inhibitors due to increased risk of infection
Kinase Inhibitors	Baricitinib 4 mg PO daily for up to 14 d Ruxolitinib 5–20 mg PO twice daily, for 14 days	**NIH Guidelines:** Baricitinib recommended in combination with remdesivir in hospitalized, nonventilated pts on supplemental O2 only if corticosteroids cannot be used (BIIa) Either baricitinib or tocilizumab is recommended in conjunction with dexamethasone in pts within 3 d of hospitalization who have rapid respiratory decompensation (admitted to ICU within 24 h and require mechanical ventilation, noninvasive ventilation, or high-flow, or have rapidly escalating O2 needs and require noninvasive ventilation or high-flow with a CRP ≥ 75 mg/L) (BIIa) Recommend against use of other kinase inhibitors outside of a clinical trial (AIII) **WHO Guidelines:** Agent not addressed **IDSA Guidelines:** Baricitinib recommended with remdesivir in pts with severe COVID-19 who cannot receive a corticosteroid (conditional recommendation, low certainty)	Substrate of CYP3A4 (minor), OAT1/3, P-gp/ABCB1 (minor)	Upper respiratory tract infections Herpes simplex and Herpes zoster Nausea Thrombosis Neutropenia, lymphopenia, anemia	Authorized for use with remdesivir under FDA EUA for pts meeting specific criteria Not recommended in patients with severe hepatic impairment Renal dosing required Drug should be discontinued if ALC <200 cells/µL, ANC <500 cells/µL, eGFR <15 mL/min/1.73 m^2^, or drug-induced liver injury develops Tablets can be dispersed in water
Interferons	Interferon alpha, interferon beta	**NIH Guidelines:** Recommends against use in pts with severe or critical COVID-19 outside of a clinical trial (AIII) Insufficient data to recommend either for or against use for the treatment of early mild or moderate COVID-19 **WHO Guidelines:** Agent not addressed **IDSA Guidelines:** Agent not addressed	No clinically significant drug interactions	Flu-like symptoms Headache Fatigue Myalgia Hepatic injury Psychiatric problems Hematologic abnormalities	Among SOT recipients, the enhancing of the immune response may result in allograft rejection and should be considered a potential risk for this population Mostly studied as nebulization for COVID-19; formulation not approved for use in US

Abbreviations: ALT, alanine aminotransferase; ANC, absolute neutrophil count; AST, aspartate aminotransferase; d, day; ECMO, Extracorporeal membrane oxygenation; eGFR, estimated glomerular filtration rate; EUA, emergency use authorization; FDA, U.S. Food and Drug Administration; GI, gastrointestinal; HBV, Hepatitis B virus; HSV, Herpes Simplex virus; IDSA, Infectious Diseases Society of America; IV, intravenously; NIH, U.S. National Institutes of Health; P-gp, P-glycoprotein; PO, per os (by mouth); pt, patient; SOT, solid organ transplant; TB, tuberculosis; ULN, upper limit of normal; WHO, World Health Organization. Data updated as of August 12, 2021. ^a^ Mutiple strategies exist. Dosing from RECOVERY Trial is listed in table. ^b^ High-titer convalescent plasma is defined as a neutralizing antibody titer of ≥250 in the Broad Institute’s neutralizing antibody assay or an S/C cutoff of ≥12 in the Ortho VITROS IgG assay.

Early administration of high titer CP appears to be more promising. In an RCT conducted by Libster and colleagues, high titer CP or placebo was given to high-risk outpatients with mild COVID-19 who were symptomatic for 72 h or less [35]. There was a 48% relative risk reduction in development of severe respiratory disease (16% of patients receiving CP versus 31% of patients receiving placebo). In an updated report from the EAP, a modest mortality benefit was noted for high titer CP (versus low titer) and earlier administration [36]. CP also appears to be beneficial in some hospitalized patients with primary or secondary immunodeficiencies [37,38,39]. In the largest retrospective study of CP treatment among SOT recipients, 8 of 13 patients had de-escalating oxygenation support at day 7; a total of 9 (69%) patients were discharged and 3 (23%) patients died at the time of the report [40]. In a review of nine other reports, the majority of 17 SOT recipients with COVID-19 had improvement in symptoms following CP administration [41].

Given these data, in February 2021 the FDA updated the EUA with two notable changes: low titer CP was no longer authorized and hospitalized patients had to be early in their disease course or have impaired humoral immunity without antibody response. Among patients without impaired immunity, consensus guidelines generally recommend against use (Table 1). For hospitalized patients with impaired immunity, the NIH guidelines state that there is insufficient evidence to recommend either for or against the use of CP; however, CP is authorized under the EUA for treatment in this population. CP may be a treatment option for SOT recipients in this setting.

### 3.3. Remdesivir

Remdesivir, an agent originally developed for the treatment of Ebola virus infection, is active against zoonotic and human coronaviruses including SARS-CoV-1 and Middle East respiratory syndrome coronavirus (MERS-CoV) [42]. Remdesivir is an adenosine nucleotide pro-drug that is intracellularly metabolized to a nucleoside monophosphate intermediate. Once phosphorylated, it acts as an analog of adenosine triphosphate for incorporation into nascent RNA chains by the viral RNA-dependent RNA polymerase with high affinity resulting in delayed chain termination and inhibition of the viral RNA-dependent RNA polymerase [43,44]. In February 2020, Wang and colleagues found the that the concentration necessary for activity against SARS-CoV-2 was likely to be therapeutically achievable in non-human primates [45]. The combination of safety and dosing data from Ebola virus studies, in addition to evidence of in vitro efficacy against SARS-CoV-2, allowed for the drug to be made available early during the pandemic for compassionate use. By May 2020, FDA issued an EUA for remdesivir based on preliminary data from the Adaptive COVID-19 Treatment Trial 1 (ACTT-1) and Gilead Sciences’ SIMPLE trials. Then, in October 2020, the drug was granted FDA approval after the final reports of the ACTT-1 and SIMPLE studies were published, along with World Health Organization (WHO) preliminary trial data.

There have been two large-scale placebo-controlled, double-blind randomized controlled trials (RCTs) of remdesivir for the treatment of COVID-19. The ACTT-1 Trial found that among hospitalized patients with COVID-19 who required supplemental oxygen or ventilatory support, a 10-day course of remdesivir reduced time to recovery compared to placebo (shorter by a median of 4 days), and clinical improvement based on an ordinal scale was higher at day 15 in patients on remdesivir compared to placebo; no mortality benefit by day 29 was observed [46]. Patients on supplemental oxygen benefitted the most; however, no benefit was observed in those on high-flow oxygen, mechanical ventilation, or ECMO. The second RCT, conducted by Wang and colleagues, found no difference in time to clinical improvement or 28-day mortality between the 10-day remdesivir and placebo arms among patients with severe COVID-19, but this study was likely underpowered [47].

Three additional large-scale open-label trials have been conducted: two trials sponsored by Gilead Sciences and The WHO Solidarity Trial. The first company sponsored trial was a RCT of patients hospitalized for COVID-19 who were on supplemental oxygen, but not mechanically ventilated, randomized 1:1 to either a 5- or 10-day course of remdesivir [48]. They found no difference between groups in clinical improvement at 14 days; however, the study lacked a control group and may have been underpowered. The second company sponsored trial was a RCT of hospitalized patients with moderate COVID-19 randomized 1:1:1 to receive a 5-day course of remdesivir, a 10-day course of remdesivir, or standard of care [49]. On day 11, patients in the 5-day remdesivir arm, but not the 10-day remdesivir arm, had better outcomes than those who received standard of care, raising questions about the clinical significance of these findings. In the third open-label RCT, the Solidarity Trial conducted by the WHO, patients hospitalized with COVID-19 of any severity were randomized to receive one of 5 options that included 5 or 10 days of remdesivir and no trial drug. When compared to its control, remdesivir did not reduce in-hospital mortality, need for ventilation, or duration of hospitalization [50].

SOT recipients and patients on immunosuppressive medications were not explicitly excluded in any of the five aforementioned trials. However, in the Solidarity Trial, patients could be excluded if they had a contra-indication to any of the treatment arms. It is plausible that SOT recipients were excluded based on immunosuppressant drug interactions with lopinavir/ritonavir, one of the treatment arms. Furthermore, in the descriptions of comorbid conditions among study subjects, none of the five trials report history of transplantation, therefore SOT recipient enrollment is unknown. Several retrospective case series of SOT recipients with COVID-19 have been conducted, but only a minority in each received remdesivir, making outcomes stratified by treatment unknown. Drug-drug interactions and adverse effects of remdesivir are detailed in the Table 1.

The evidence of clinical benefit of remdesivir is felt to be strongest among patients hospitalized for COVID-19 who require supplemental oxygen only. As such, the NIH guidelines most strongly recommend the use of remdesivir, generally with dexamethasone, for treatment of COVID-19 in this population [9]. The Infectious Diseases Society of America (IDSA) guidelines also recommend use of remdesivir among patients who require supplemental oxygen [33]. They also recommend its use among patients mechanically ventilated or on extracorporeal membrane oxygenation (ECMO). In contrast, the WHO guidelines recommend against the use of remdesivir in patients with COVID-19 due to lack of evidence of improved outcomes [32]. Full society recommendations are listed in the Table 1.

### 3.4. Corticosteroids

Data from the SARS-CoV-1 and MERS outbreaks suggested corticosteroids were associated with delayed viral clearance, which initially cast doubt on their use for COVID-19 [51]. Additionally, corticosteroids were associated with worse outcomes, including death, in a trial of patients with severe pneumonia due to influenza viruses [52]. Conversely, a meta-analysis found that corticosteroids reduced mortality and duration of ventilation compared with placebo among patients with ARDS. As such, suppression of various cytokines garnered interest to decrease the CRS associated with COVID-19 [53].

Several notable studies of corticosteroids for the treatment of COVID-19 have been published. Data from the RECOVERY Trial have been the strongest proponent for the use of corticosteroids. In this controlled, open-label trial, patients hospitalized with COVID-19 either received dexamethasone 6 mg daily for up to 10 days or standard of care [54]. Among patients requiring supplemental oxygen with or without invasive mechanical ventilation, 28-day mortality was significantly lower among those receiving dexamethasone versus standard of care [54]. This effect was not preserved among patients not receiving respiratory support. Data from other studies are more conflicting. In the CAPE COVID study, which was stopped after the results of RECOVERY became available, there was no difference in mortality or persistent respiratory support among intensive care unit (ICU) patients receiving low-dose hydrocortisone or placebo [55]. Likewise, in the CoDEX trial of higher dose dexamethasone among patients who were mechanically ventilated, there were no differences in 28-day mortality, but the dexamethasone arm had more ventilator-free and alive days than the placebo arm [56]. In the REMAP Trial, ICU patients given hydrocortisone had higher odds of improvement in organ support-free days versus placebo [57]. The RECOVERY and CoDEX trials do not provide details on participants with immunosuppressive conditions or on immunosuppressive medications; in each the CAPE COVID and REMAP trials, less than 10 patients with these conditions were included in corticosteroid arms. As such, the effect of corticosteroids for COVID-19 in the SOT recipient population is unknown.

Consensus guidelines currently recommend the use of dexamethasone among patients requiring supplemental oxygen, including for those who require oxygen via a high-flow device, invasive mechanical ventilation, and ECMO (Table 1). Although not extensively studied, the combination of corticosteroids and remdesivir has been recommended for hospitalized patients requiring supplemental oxygen, but not for those requiring invasive mechanical ventilation (Table 1).

If SOT recipients are receiving corticosteroids prior to COVID-19 illness, it is likely reasonable to continue therapy. Information regarding drug-drug interactions, adverse effects, and special considerations can be found in the Table 1.

### 3.5. Interleukin (IL)-6 Inhibitors (Sarilumab, Tocilizumab, Siltixumab) and IL-1 Inhibitor (Anakinra)

Increased circulating levels of IL-1 and IL-6 have been noted in severe SARS-CoV-2 infection. As such, interest has been directed at modulation of these levels. IL-6 has been targeted when managing CRS in relation to chimeric antigen receptor (CAR) T cell therapy [58]. There are currently several IL-6 inhibitors on the US market: anti-IL-6 receptor monoclonal antibodies such as sarilumab and tocilizumab, and anti-IL-6 monoclonal antibodies such as siltuximab. Tocilizumab is FDA approved for the indication of CAR T-cell-associated CRS, and anakinra, an IL-1 receptor antagonist, has also been used off-label for this indication.

Tocilizumab and sarilumab are humanized monoclonal antibodies that bind to membrane-bound and soluble forms of human IL-6 receptors, competitively inhibiting IL-6 binding and signal transduction. Early trials of tocilizumab were limited by small sample size leading to low power, use across a spectrum of COVID-19 illness, and absence of dexamethasone use. More recently, larger RCTs have been conducted investigating treatment with tocilizumab. Collective data from these RCTs appeared to signal that tocilizumab was most beneficial among patients with progressive moderate or severe COVID-19 who had not yet required or recently required invasive mechanical ventilation [59,60,61,62]. The largest tocilizumab RCTs, RECOVERY and REMAP-CAP, confirmed the benefit of the drug among the sickest patients with COVID-19, most of whom (>80%) were receiving concomitant dexamethasone [63,64]. In REMAP-CAP, patients with COVID-19 who were admitted to an ICU and began requiring respiratory support (invasive or noninvasive mechanical ventilation) or cardiovascular support within the previous 24 h were randomized to receive tocilizumab (*n* = 353), sarilumab (*n* = 48), or placebo (*n* = 402) [64]. Patients receiving tocilizumab or sarilumab had more organ support-free days (median of 10 days for tocilizumab and 11 days for sarilumab) versus placebo (median of 0 days) and improved 90-day survival (hazard ratio of pooled IL-6 inhibitors compared with control of 1.61). In RECOVERY, over 4000 hospitalized patients with COVID-19 with hypoxia and a C-reactive protein (CRP) ≥75 mg/L were randomized to receive tocilizumab or standard of care [63]. Mortality at 28 days was lower in the tocilizumab arm versus the usual care arm (29% vs. 33%) and the proportion of patients discharged alive at 28 days was higher in the tocilizumab arm versus the usual care arm (54% vs. 47%); however, there was no difference in mortality or 28-day discharge in a subgroup analysis of patients requiring mechanical ventilation at baseline.

The safety and efficacy of interleukin inhibitors among SOT recipients is less well described. In an observational cohort of 80 kidney transplant recipients who received tocilizumab for severe COVID-19, the mortality rate at the time of publication was 32.5% [65]. CRP levels decreased following tocilizumab and the decrease positivity correlated with survival. In a cohort of 117 SOT recipients, 29 who received tocilizumab were compared to matched controls who did not receive tocilizumab [66]. There was no difference in mortality. Secondary infections occurred in over one third of patients in the tocilizumab group, but this was not significantly higher than the matched control group. The most common secondary infections in the tocilizumab group were CMV reactivation (17%), bacterial pneumonia (10%), and blood stream infections (7%). There were also no differences in rates of venous thrombosis, cerebrovascular events, and bowel perforation. The authors concluded that tocilizumab is likely safe and has minimal impact on the net state of immunosuppression among SOT recipients who are receiving corticosteroids and are critically ill.

The NIH guidelines currently recommend use of tocilizumab in combination with dexamethasone for recently hospitalized patients admitted to an ICU within the previous 24 h who require invasive mechanical ventilation, noninvasive ventilation, or high-flow nasal canula oxygen, or patients not admitted to an ICU who have rapidly increasing oxygen needs and a CRP ≥ 75 mg/L (Table 1). The IDSA guidelines recommend tocilizumab more liberally to also include patients with progressive severe COVID-19 requiring supplemental oxygen with a CRP ≥75 mg/L. The use of sarilumab and siltuximab is not currently recommended due to the low number of patients receiving sarilumab in RCTs and limited data on siltuximab. Data on anakinra is limited to case series data and the NIH guidelines do not recommend for or against its use at this time (Table 1).

Importantly, the NIH guidelines state that tocilizumab should be avoided in patients with significant immunosuppression [9]. Outside of COVID-19, cumulative safety data from several phase 3 trials of tocilizumab for rheumatoid arthritis found a serious infection and opportunistic infection rate of 4.7 and 0.23 per 100 patient years, respectively [67]. In a cohort of 36 kidney transplant recipients treated with monthly tocilizumab for antibody-mediated rejection, 5 patients had cytomegalovirus infection, 2 patients had polyoma BK infection, 1 patient had a skin condition related to polyoma virus, and 7 patients had bacterial infection [68]. However, these data may not represent infection risk among patients receiving only one dose of tocilizumab for COVID-19. More reassuringly, a recent study of immunocompromised patients with CAR T-cell-associated CRS treated with 1 to 3 doses of tocilizumab (n = 166) did not have an increase in infectious risk compared with those not given tocilizumab [69]. The decision to use IL-6 inhibitors in SOT recipients with COVID-19 should be made carefully, as the efficacy and safety in this population are not well established.

### 3.6. Kinase Inhibitors

There are two classes of kinase inhibitors that are FDA-approved for certain rheumatologic or oncologic indications that have also been proposed for the treatment of COVID-19: Janus Kinase (JAK) inhibitors (baricitinib, ruxolitinib, and tofacitinib) and Bruton’s tyrosine kinase (BTK) inhibitors (acalabrutinib, ibrutinib, and zanubrutinib). These kinase inhibitors interfere with intracellular signaling pathways that lead to immune activation and inflammation [70]. In addition to the ability to inhibit a variety of proinflammatory cytokines, some kinase inhibitors may also reduce viral entry and intracellular virus particle assembly [70].

Of the kinase inhibitors, randomized clinical data exists only for baricitinib and ruxolitinib. In animal models, baricitinib inhibited inflammatory cell recruitment to the lungs, as well as suppressed proinflammatory cytokines, which limited the severity of SARS-CoV-2 infections [71]. In the ACTT-2 Trial, over 1000 hospitalized patients were randomized to receive remdesivir plus either baricitinib or placebo. The primary outcome, time to recovery, was significantly reduced with combination treatment, with the most pronounced effect observed among patients receiving high-flow oxygenation or noninvasive ventilation. Secondary outcomes showed combination therapy was associated with better clinical status at day 15 in the subgroup requiring high-flow oxygen or noninvasive mechanical ventilation [72]. In ACTT-2, patients receiving corticosteroids at enrollment were excluded, thus prohibiting an analysis on the effects of baricitinib in combination with corticosteroids. Based on this data, baricitinib was authorized under an EUA for the treatment of COVID-19 in combination with remdesivir for hospitalized patients on supplemental oxygen, invasive mechanical ventilation, or ECMO. Similar observations were made in the COV-BARRIER study, which compared baricitinib alone with placebo [73]. Over 90% of patients in the study received concomitant dexamethasone and baricitinib, which proved most useful among patients receiving high-flow oxygen or noninvasive mechanical ventilation. Data assessing ruxolitinib for treatment of SARS-CoV-2 includes a randomized trial of 43 participants with severe disease assigned to standard of care with or without ruxolitinib [74]. Ruxolitinib treatment was not associated with faster clinical improvement [74].

Data from the COV-BARRIER study led the NIH to recommend either baricitinib or tocilizumab in combination with dexamethasone alone or dexamethasone plus remdesivir for recently hospitalized patients who have rapidly escalating oxygen need, require high-flow oxygen or noninvasive mechanical ventilation, and have high markers of inflammation (Table 1). There is no data on use of baricitinib for the treatment of COVID-19 among SOT recipients. The NIH guidelines also recommend against use of other JAK inhibitors and BTK inhibitors outside of a clinical trial.

### 3.7. Others

Other repurposed drugs such as lopinavir-ritonavir, hydroxychloroquine, chloroquine, azithromycin, and ivermectin were found to have activity in vitro against SARS-CoV-2. However, these drugs have not been proven to be effective for the treatment of COVID-19 and are not currently recommended. Other immunomodulators such as intravenous immunoglobulin (IVIG) and interferons have also been suggested as treatments for COVID-19. Interferons were shown to have no benefit among patients with severe or critical COVID-19 and there is a lack of data on the use of IVIG for COVID-19 [50]. Additionally, there have been previous reports of allograft rejection with interferon therapy.

## 4. Vaccines

Development and administration of a safe and highly effective vaccine is an ultimate priority for prevention of COVID-19 and controlling the COVID-19 pandemic. However, vaccine immune response in SOT recipients is not as well understood, as immunocompromised individuals have been largely excluded from studies of SARS-CoV-2 vaccines. Currently in the US, three efficacious and safe vaccine products are available for administration. Two of these, BNT162b2 and mRNA-1273, are lipid nanoparticle-formulated, nucleoside-modified RNA vaccines encoding the SARS-CoV-2 prefusion full-length S protein of SARS-CoV-2, while Ad26. COV2.S is a recombinant, replication-incompetent human adenovirus vector encoding full-length SARS-CoV-2 spike protein [75,76,77]. They have been shown to be safe with respective efficacies of 95%, 94.1%, and 66.1% in adult patients [75,76,77]. Outside of the US, the AZD1222 and rAd26 and rAd5 (Sputnik V) replication-incompetent adenovirus vector vaccines, NVX-CoV2373 recombinant subunit adjuvanted protein vaccine, and Covaxin inactivated coronavirus vaccine demonstrated favorable safety and efficacy profiles. In healthy adults, robust CD4+ and CD8+ T cell responses and strong antibody responses develop after vaccination [78]. However, these responses may not be representative of the SOT recipient population subject to cell-mediated immunosuppression, and lower immune response rates to other vaccines have been described [79].

In a multicenter prospective cohort study of 658 SOT recipients vaccinated with a two-dose mRNA vaccine series, 357 (54%) were found to have a measurable antibody to the receptor-binding domain of the SARS-CoV-2 S protein at a median of 29 days after the second vaccine dose [80]. Notably, of the SOT recipients on antimetabolite maintenance immunosuppression, 57% showed no antibody response after completing the two-dose vaccine series, while for SOT recipients not receiving antimetabolites, only 18% had no antibody response after completing the vaccine series. Only 15% of SOT recipients had detectable antibody response at a median of 21 days after the first dose [80]. Other data have been published that shed light on vaccine humoral response in SOT recipients. Grupper et al. compared the anti-S protein IgG antibody response in 136 kidney transplant recipients to 25 controls at 10 to 20 days after completing the BNT-162b2 vaccine series [81]. The kidney transplant recipients were often on a calcineurin inhibitor (90.4%), low-dose steroids (88.9%), and mycophenolate containing product (76.4%), while the control had no immunosuppression exposure. Of the transplant group, only 37.5% showed antibody response, while 100% of the control group showed antibody response (*p* < 0.001). Use of mycophenolate containing products, recent high dose steroids, use of three immunosuppressing drugs, and older age increased risk for lack of antibody response. A similar design was used to assess SARS-CoV-2 IgG S protein and nucleocapsid antibody response rates to BNT162b2 in 80 liver transplant recipients, in which only 47.5% of the transplant group showed antibody response, and liver transplant recipients with antibody response had lower titers compared to healthy controls [82]. In 101 kidney transplant recipients treated with belatacept, of which 78.2% were also treated with mycophenolic acid, only 34.7% developed anti-spike antibodies, and 30.4% developed T cell response measured by SARS-CoV-2 specific T cell production of IFN-γ at 1 month following completion of the vaccine series [83].

Further studies are needed to elucidate T and B cell immune responses to COVID-19 vaccine in the SOT population, to assess not only vaccine efficacy in disease prevention, but also protection against severe COVID-19 in SOT recipients, and to assess vaccination response longevity. Absent or low antibody response rates to vaccines are concerning but do not suggest clinical ineffectiveness, as further studies describing prevention of severe disease and effect on clinical outcomes of breakthrough cases are needed. Until more data is available, the American Society of Transplantation advises pre-transplant vaccination of all SOT candidates when feasible, continued SARS-CoV-2 vaccination for SOT recipients and their household members and caregivers, continuation of a stable immunosuppression regimen, and continued protective mitigation measures [84]. 

## 5. Conclusions

At this time, there is minimal data on clinical outcomes among SOT recipients treated with differing therapies for COVID-19. As such, consensus guidelines recommend that clinicians should treat SOT recipients with COVID-19 using a similar approach to nontransplant patients. In addition to proven treatments such as dexamethasone, remdesivir, tocilizumab, and baricitinib, the use of monoclonal antibodies and convalescent plasma in particular warrant further investigation among SOT recipients, as these patients are possibly less likely to mount effective cell-mediated and adaptive immune responses. Additional considerations for SOT recipients with COVID-19 include careful adjustment of immunosuppressive medications in conjunction with a transplant specialist, assessments of drug-drug interactions, and review of overlapping medication toxicities. Further data is needed to individualize COVID-19 treatment among SOT recipients with varying degrees of disease severity.
